# Prediction of Filamentous Sludge Bulking using a State-based Gaussian Processes Regression Model

**DOI:** 10.1038/srep31303

**Published:** 2016-08-08

**Authors:** Yiqi Liu, Jianhua Guo, Qilin Wang, Daoping Huang

**Affiliations:** 1 School of Automation Science & Engineering, South China University of Technology, Wushang Road, Guangzhou 510640, China; 2 Advanced Water Management Centre, The University of Queensland, St. Lucia, Brisbane QLD 4072, Australia

## Abstract

Activated sludge process has been widely adopted to remove pollutants in wastewater treatment plants (WWTPs). However, stable operation of activated sludge process is often compromised by the occurrence of filamentous bulking. The aim of this study is to build a proper model for timely diagnosis and prediction of filamentous sludge bulking in an activated sludge process. This study developed a state-based Gaussian Process Regression (GPR) model to monitor the filamentous sludge bulking related parameter, sludge volume index (SVI), in such a way that the evolution of SVI can be predicted over multi-step ahead. This methodology was validated with SVI data collected from one full-scale WWTP. Online diagnosis and prediction of filamentous bulking sludge with real-time SVI prediction was tested through a simulation study. The results showed that the proposed methodology was capable of predicting future SVIs with good accuracy, thus providing sufficient time for predicting and controlling filamentous sludge bulking.

Eutrophication caused by excess wastewater discharge is recognized as a serious water pollution problem worldwide. To prevent water pollution, biological wastewater treatment has been widely adopted to remove pollutants. In terms of its economical and technical feasibility, activated sludge process (ASP) is the most commonly used biological wastewater treatment process to remove organic matter and nutrients (mainly nitrogen and phosphorus). The successful operation of ASP mainly relies on efficient biological conversions in bioreactors and normal sludge separation in secondary clarifiers. Filamentous bacteria are normal components of activated sludge biomass, where the existence of a fraction of filamentous bacteria is important and helpful to form flocs by serving as the floc-backbone for other bacteria to attach^1^. However, filamentous bulking sludge, a term used to describe the excess proliferation of filamentous bacteria, often results in deteriorating sludge settleability, poorer operational performance and higher treatment cost^2^,^3^. Filamentous bulking sludge is considered the most serious problem usually happening in WWTPs adopting ASP. It is documented that more than 50% of WWTPs encounter the filamentous bulking sludge problem worldwide^3^.

In order to prevent from serious deterioration of sludge settleability, predictive models have been proposed to achieve early warning for filamentous sludge bulking. In terms of underlying model development fundamentals, predictive models can be divided into three categories, i.e., mechanistic, gray-box and black-box models^4^. The mechanistic models are often used to achieve optimization and control of process. For example, the activated sludge model family^5^ can provide a comprehensive description of the significant biological processes of the ASP system, while a secondary settling tank model^6^ can provide more realistic predictions of the sludge settleability performance. Compared to mechanistic models, gray-box modeling approaches are relatively simple for the design of predictive models, since a reduced number of parameters will be estimated by statistical or mathematical techniques. Predictive methodologies based on black-box models also gains popularity resulting from the fact that they do not require detailed understanding of the system^7^. In addition, black-box models are cost-effective to perform prediction, which attempt to automatically capture the dominant processes and to link input to output variables, consequently being seen as an alternative when mechanistic models are not available or not valid. To predict filamentous sludge bulking, few linear black-box models, such as Auto-Regressive and Moving Average (ARMA)^8^, Hidden Markov model^9^ and partial least squares (PLS)^10^ were performed. However, due to strong non-linearity, predictions using such linear models (e.g. ARMA and PLS) often deviate significantly from the real state of the WWTP. Thus, nonlinear models are proposed to deal with these problems, including the nonlinear PLS method^11^, artificial neural networks^12^ and the support vector machine (SVM) based regression method^13^,^14^. Although these nonlinear models exhibit advantages to predict filamentous sludge bulking, building a high performance prediction model is very laborious, since input variables and samples for model construction have to be selected carefully and parameters have to be tuned appropriately. Particularly for the artificial neural networks^15^,^16^, the number of layers and the number of neurons for each layer should be selected carefully by trial and errors. Furthermore, the computational intensity is prohibited for most of cases, e.g. the number of layers being more than six.

To predict filamentous sludge bulking timely, the model needs not only to capture strongly nonlinear effects of operational conditions on sludge settleability, but also to be robust for the presence of uncertainty and noise in WWTPs. Building models for activated sludge systems purely based on a theoretical understanding of underlying physical or biochemical principles can be unfeasibly complex and requires a large number of simplifying assumptions. Additionally, uncertainty in the WWTP would have negative influences on a certain decision making for a reliable operation of the system. If ignoring uncertainty or relying on the expectation of uncertainty, the prediction could be poor and result in deviation of fault diagnosis or prognosis. Gaussian Process for regression (GPR) model is a new proposed distribution-driven methodology, which is not only able to model dynamic processes of both linear and nonlinear systems, but also to generate predicted distribution (interval prediction), rather than point prediction, to facilitate our decision making for filamentous bulking^17^. Traditionally, predicted models output a bare prediction without any associated confidence values and hence have to rely on the previous experience or relatively loose theoretical upper bounds on the probability of error to gauge the quality of the given prediction. On the contrary, resulted intervals from the GPR model would become wide or narrow to indicate how confident it is for the predicted values. Also, through the choice of covariance function, a wide range of modeling assumptions would be expressed to be able to approach different operational states of WWTP.

Generally, black-box models are able to serve as the basis to assist in fault diagnosis. The task of fault diagnosis is mainly to identify the abnormal states and recognize them as early as possible, thus enabling prevention of emergent accidents and reducing the cost of downtime maintenance. However, one-step (OS) ahead prediction does not provide sufficient time for sludge settleability recovery. Therefore, it is imperative to recognize future emergent events in advance as early as possible. One of plausible ways is to apply multi-step (MS) ahead prediction, which is able to predict the progress of the filamentous bulking for a long-term operation MS ahead prediction would also add further complexity of uncertainty description for a GPR model, as the propagation of uncertainties is calculated recursively as prediction moves ahead. So far, few studies have devoted to fault diagnosis and fault prognosis (fault prediction) with MS ahead prediction in bio-chemical processes^18^. In particular, few attempts based on MS ahead prediction are used for filamentous sludge bulking fault diagnosis and prognosis.

The objective of this study is to develop a state-based GPR model aiming to detect and provide a warning for the prevention of sludge bulking in advance. The contributions of this paper are summarized as follows. Firstly, this study is the first attempt to develop a state-based GPR model to characterize for the evolution of filamentous sludge bulking. Given different degrees of severity during the evolution of filamentous sludge bulking, two types of GPR models, i.e., OS ahead prediction for normal state and MS ahead predictions after recognition of filamentous sludge bulking, are constructed in this model, respectively. Further, to coordinate the both of OS and MS GPR models as well as different states concerning the evolution of filamentous sludge bulking, hybrid automata is introduced, which is able to characterize the transitions of OS and MS GPR models smoothly. Secondly, since distribution-driven model, GPR, is used for prediction, uncertain factors contributing to filamentous sludge bulking can be accounted, thus providing distribution-based prediction, rather than point prediction for filamentous sludge bulking, which would alleviate the false alarms properly during the fault diagnosis and prognosis processes. Finally, considering it is difficult to define an accurate threshold to distinguish different sludge bulking state, we proposed to use an interval fault diagnosis limit to envelop the uncertainties resulting from the model parameters and external disturbances (variations of wastewater components and weather influence).

## Results

### Development of predictive model for fault diagnosis and prognosis of filamentous bulking

In practice, Sludge Volume Index (SVI) is an empirical measurement used to characterize the sludge bulking problem^19^. The SVI is able to represent different typical states of filamentous bulking sludge, when SVI reaches the pre-specified level (in most cases SVI of 150 mL/g as a threshold value). Some different values for SVI of 100, 180, even 280 mL/g are also used as a threshold value to distinguish filamentous bulking sludge with good sludge settleability^20^. Definition of an accurate threshold to indicate filamentous sludge bulking is still an open issue. In the previous study^21^, three different sludge states are defined in terms of SVI values: normal state with SVI of 0–150 mL/g, limited filamentous bulking sludge with SVI of 150–250 mL/g and serious filamentous bulking sludge with SVI of more than 250. However, by taking into account the negative influence of uncertainties (such as variations of wastewater components, model parameters and weather influence, render accurate measurement of SVI impossible), ±10% is added to the control limit of limited filamentous bulking sludge in this study. On the contrary, due to severity of serious filamentous bulking sludge, the hard control limit of 250 mL/g is used. Consequently in this study, four states (State 1: SVI of 0–135 mL/g, normal state; State 2: SVI of 135–165 mL/g, limited filamentous bulking sludge pre-caution; State 3: SVI of 165–250 mL/g, limited filamentous bulking sludge; State 4: SVI of more than 250 mL/g, serious filamentous bulking sludge) are defined in our models.

**Figure 1:**
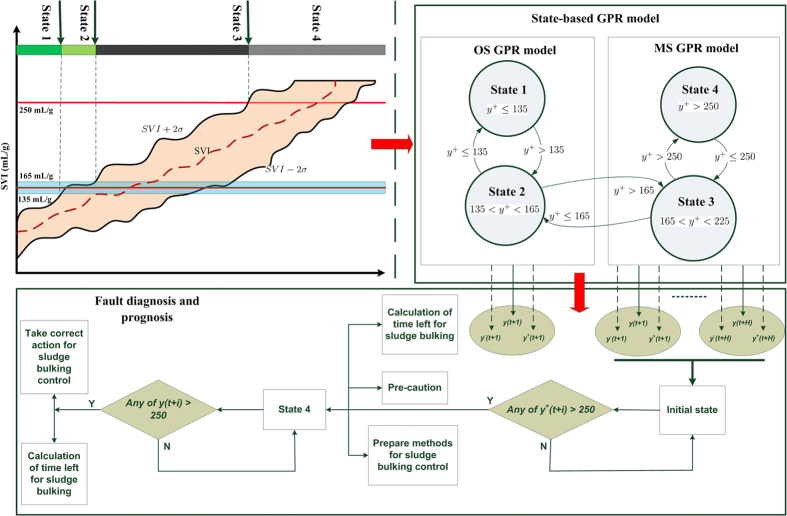
The schematic of construction of prediction model and fault prognosis (SVI: sludge volume index; OS GPR: One-step Gaussian Processes Regression; MS GPR: Multi-step Gaussian Processes Regression; *y*: SVI; *y*^−^: *SVI* − 2*σ*; *y*^+^: *SVI* + 2*σ*).

To characterize different states of SVI, the construction of GPR models becomes important, since it serves as the basis for the state-based GPR. Firstly, the OS GPR model is built for States 1 and 2 as shown in Fig. 1. The most important part of the OS GPR model is to identify the unknown parameters by maximize the corresponding likelihood function. Then, by supplying the new coming data points x^*^, the prediction values of SVI and their corresponding variances, *SVI *− 2*σ* and *SVI *+ 2*σ* can be obtained. Secondly, the MS GPR model is built for States 3 and 4 as well (Fig. 1). Different from the OS GPR model, only time series data of SVI is selected due to recursive MS prediction strategy applying to perform the MS GPR model. By performing the MS GPR model, prediction values of SVI and their corresponding variances, *SVI *− 2*σ* and *SVI *+ 2*σ* over the entire prediction horizon, *H*, can be derived. Thirdly, both of OS GPR model and MS GPR are further served as the basis for a hybrid automata (the details can be found in the section of Methods). The hybrid automaton is a mathematical model for precisely describing systems in which digital computational processes interact with analog physical processes^22^,^23^. Differently, this paper implements hybrid automata for the accurate characterization of interactions between OS ahead and MS ahead prediction. To date, this has not been applied to filamentous bulking sludge prediction, even though the use of hybrid automata for this purpose in chemical industries is widespread. The hybrid automata for SVI is shown as Fig. 1 to coordinate different states. The variable *y*, *y*^*−*^ and *y*^+^ represent the SVI, *SVI *− 2*σ* and *SVI *+ 2*σ*, respectively. Hereby, *σ* is the variance of predicted SVI obtained from the GPR model, representing the effect resulting from the uncertainty (model parameters, uncertainty for collected data, and significant disturbances of a WWTP). The reason why *y*^+^ is used to recognize the filamentous sludge bulking occurrence is the fact that *y*^+^ is prone to capture the worst case and enable the prevention of filamentous sludge bulking in advance. In control States 1 and 2, filamentous bulking sludge did not occur yet, SVI and its corresponding uncertain boundary can be obtained for OS ahead prediction using the developed GPR (the details can be found in Section Methods), respectively. In control mode State 1, the SVI is on the normal state. According to the jump condition *y*^+^ > *135* (limited filamentous bulking sludge lower limit), the SVI is still calculated by the same GPR model as State 1, but the state of limited filamentous bulking sludge pre-caution should be declared to indicate the potentials of limited filamentous bulking sludge. On the contrary, the SVI will jump back state 1 if *y*^+^ < *135*. Once the *y*^+^ runs over 165, the MS GPR model will be triggered to make multi-step ahead prediction. This is mainly due to the significant potentials to transit from limited filamentous bulking sludge to severe filamentous bulking sludge. States 3 and 4 jump on the basis of their corresponding jump conditions. Even though *y*^+^ generated from GPR models is able to provide a pre-caution in advance and to alleviate the deviations of predicted values, *y*. The value of *y* can be used to further confirm the occurrence of sludge bulking.

### A Case study-filamentous sludge bulking diagnosis and prognosis in a full-scale WWTP

**Figure 2:**
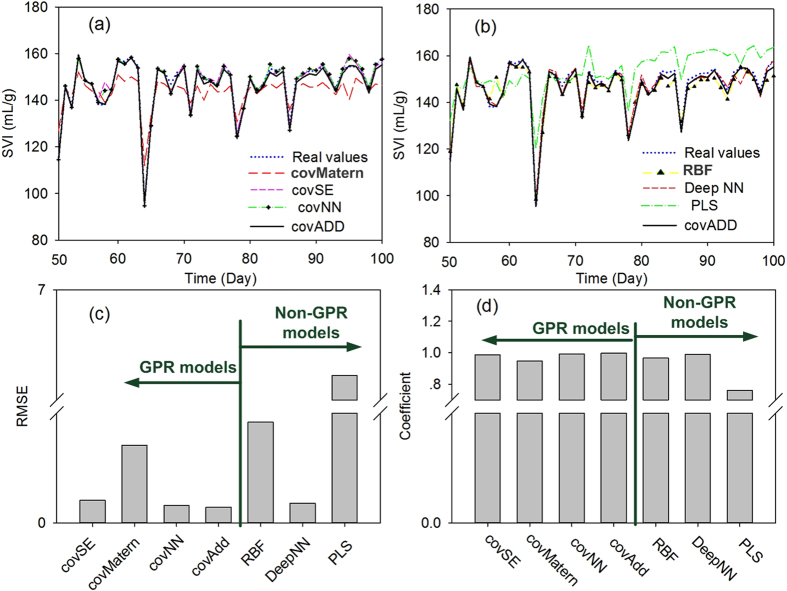
Schematic diagram of a full-scale oxidation ditch process.

The presented case is a full-scale WWTP (Beijing, China), which mainly treated municipal wastewater with an Oxidation ditch (OD) process. OD process is a modified activated sludge biological treatment process that utilizes long solids retention time (SRT) to achieve good nitrogen removal performance. Figure 2 shows a schematic of the reactor for the full-scale WWTP. In this plant, the average influent flow was about 170,000 m^3^/d, with an average OD hydraulic retention time (HRT) of 16.5 h. SRT was kept 15–22 d by withdrawing sludge from the second settler. Due to low COD loading rate (<0.25 kgCOD/kgMLSS/d), the occurrence of filamentous bulking sludge was observed in this plant. The phenomenon of bulking sludge lasted for about half a year. The influent characteristics, operation conditions and the evolution of SVI were recorded during the period of filamentous sludge bulking. These data were used to develop and validate the GPR model in this study.

In order to select the most relevant variables as inputs for the OS-GPR model, the Variable Importance in Projection (VIP) method together with the mechanical knowledge is used^24^. Eight process variables are selected as model inputs for the OS-GPR model, as listed in Table 1. A total of 213 data points are collected from the full-scale operational observations. Of these samples, 50 were utilized for OS-GPR model training, while the remaining 50 samples before limited filamentous bulking occurrence were used to test the performance of the OS-GPR-based prediction. However, once the SVI transits to limited filamentous bulking, MS-GPR model is trained using the data before the occurrence of limited filamentous bulking. Since the transition of SVI is from the 101^st^ data, all of the previous data are used for MS-GPR model training. The remaining data are then left for MS-GPR model and fault prognosis testing. It should be noted that different from OS-GPR model usage of eight variables as inputs, MS-GPR model is constructed based on time series data, i.e., the most recent lagged SVI (More details see Table 1). The dimension selection for MS-GPR modeling inputs is achieved by performing Auto Correlation Function (ACF) and Partial Auto Correlation Function (PACF)^25^.

### Prediction of limited filamentous bulking with one-step ahead prediction models

In this paper, the Root Mean Square Error (RMSE) and coefficient (*r*) were used to access the prediction performance of inferential model. The Root Mean Square Error (RMSE criterion is defined as follows for quality comparisons of different methods


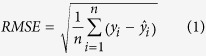


where *y*_*i*_ and 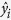 are the measured and prediction values, respectively, *n* represents the number of sample points.

**Figure 3:**
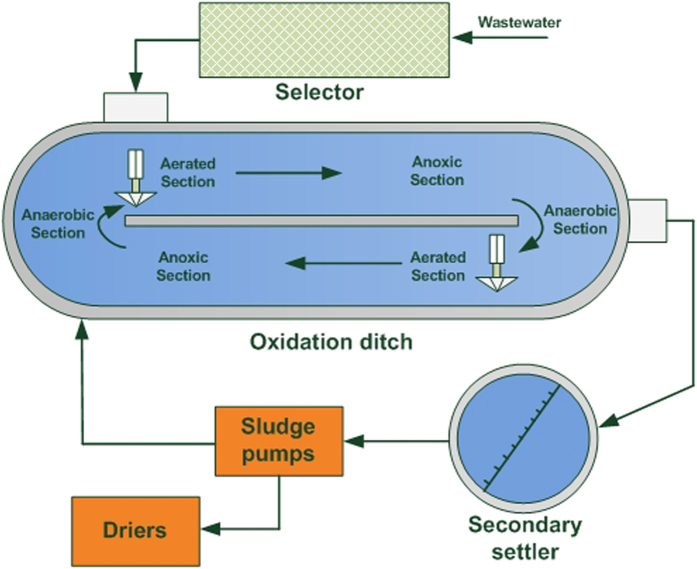
Comparisons of Gaussian processes for micro sludge bulking diagnosis with different covariance functions (covSE: Squared-Exp kernel; covNN: Neural Network kernel; covMatérniso: Matérniso kernel; covAdd: Addictive kernel) and other models (RBF: Radical Basis Function; DeepNN: Deep Neural Network; PLS: Partial Squares Least; More details about the Kernel can see Supplementary Information).

The performance of four GPR models with different Covariance is shown in Fig. 3(a), suggesting that the GPR model with additive Covariance achieved the best performance in terms of RMSE (Fig. 3(c)) and *r* (Fig. 3(d)). The reason mainly lies on the fact that the additive Covariance is more flexible, thus being able to approach strong non-linear relationship and describe the non-local integrations of the data. In contrast, other GPR models are only limited to one type Covariance, thus being unable to track some data with different Covariance shape. In order to illustrate the efficiency of GPR model with addictive Covariance, the profiles of SVI prediction are presented in Fig. 3(b) compared with other models, suggesting that GPR model with addictive Covariance still achieves the best performance in terms of RMSE (Fig. 3(c)) and *r* (Fig. 3(d)). It is obvious that, due to being unable to capture the nonlinearity of SVI, PLS gains the worst performance, whereas the nonlinear model RBF achieved a relative better performance. In addition, Deep Neural Network, which is a new algorithm to deal with strong nonlinearity using more layers structure^26^, performed better fairly. However, its computational intensity prohibits its widely use, which is five times than the GPR model with addictive Covariance. The presented algorithms’ parameters are set up as Table S1 (Supporting Information).

### Fault prognosis with MS ahead prediction models

**Figure 4:**
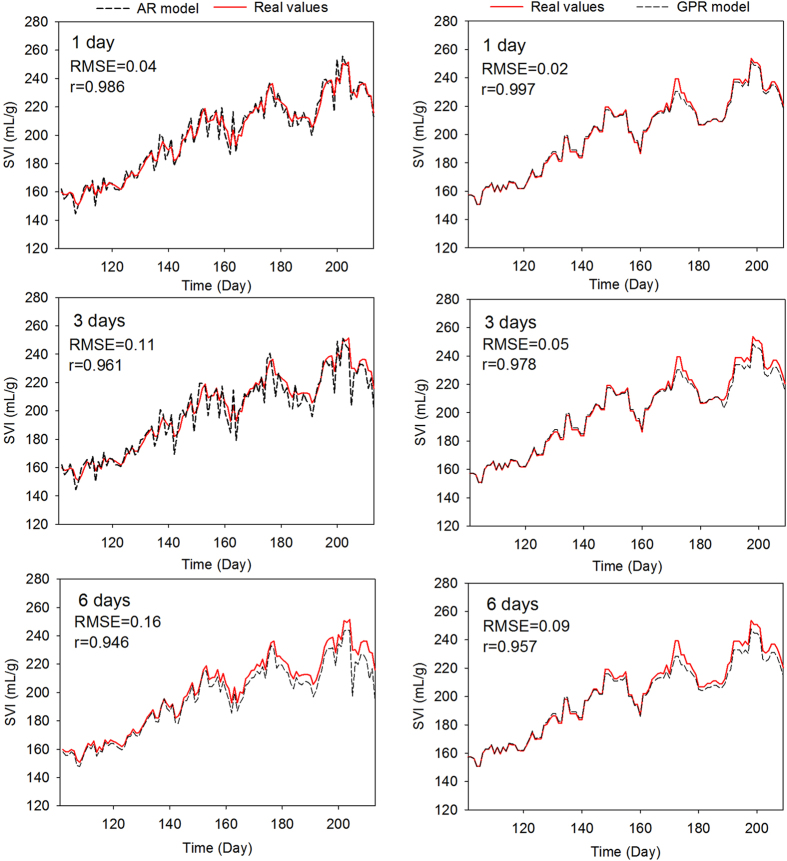
Comparisons of prognosis with different multi-steps ahead prediction models for serious sludge bulking diagnosis.

In order to account for evolution of SVI, OS prediction is not capable of providing sufficient time for filamentous sludge bulking control. Thus, once the *y*^+^ crosses control limit 165 mL/g, MS-GPR model is triggered immediately. To evaluate the performance of fault prognosis, the ARMA model, which is widely used for multi-steps-ahead prediction, is tested for 1, 3 and 6 days ahead predictions firstly. Obviously, the ARMA model is able to capture the propagation of fault, even though a bit shifts is happening as more days ahead prediction. Also, due to the noise sensitivity, the ARMA model exhibits a significant fluctuation and thus further deteriorates the performance of corresponding models. As compared to the ARMA model, the GPR model is also presented. The time lag of GPR is set to be 7 days by considering the correlation between few recent lagged samples. As shown in Fig. 4 (right), the GPR model with additive Covariance achieves a relative better performance for three kinds of scenarios (1, 3 and 6 days ahead predictions) in terms of RMSE and *r*.

### Fault prognosis with uncertainty analysis

**Figure 5:**
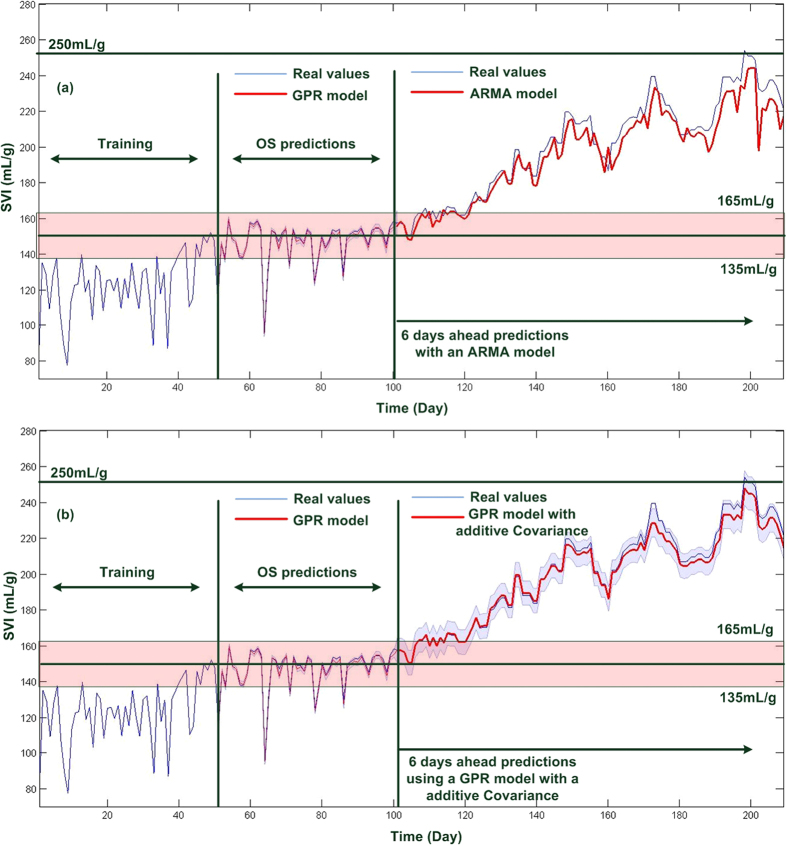
Fault alarms and model uncertainty analysis.

Since uncertainty in a WWTP might be resulted from many factors such as equipment degradation, plant model mismatch and other disturbances, decision-making under uncertainty is not trivial if it is desired to fulfill reliability requirements and quality standards. To reduce the false alarms and ensure robust control limit for diagnosis in our diagnosis methodologies, we relaxed the limited filamentous bulking control limit to 150 ± 15 mL/g, rather than 150 mL/g exactly. Such relaxation represents 90% confidence on the diagnosis results. It is obvious that the boundaries of GPR models during the OS and MS perdition periods are capable of enveloping the variations of SVI (Fig. 5). Since the OS ahead prediction has been in the envelop of 150 ± 15 mL/g, the limited filamentous bulking pre-caution is forwarded. Sequentially, due to the use of 6 days ahead predictions after recognizing the ending of limited filamentous bulking, the boundary of the MS-GPR model become wider, representing less confident on the prediction results. The wider boundary is, in turn, able to envelope the true values properly. In these methodologies, the worst case strategy is considered. Once the upper boundary of OS-GPR model crosses over 165 mL/g, MS-GPR model is triggered to perform multiple ahead predictions to indicate how far it is from the serious filamentous bulking pre-caution control limit, 250 mL/g (Fig. 5). Figure 5 also suggests that, even though the MS-GPR model cannot tract the evolution of SVI exactly after day 190, the uncertainty envelop is able to compensate such deviations and facilitate the fault prognosis properly.

## Discussion

The present work investigated the use of GPR model for the fault diagnosis and prognosis of filamentous sludge bulking in activated sludge process. The OS-GPR and MS-GPR models are coordinated by hybrid automata to provide a simple, but powerful tool to predict the evolution of SVI. This, along with uncertainty information that was generated from a GPR model by nature, supported the design of fault diagnosis and prognosis of filamentous bulking, yielding excellent performance. To the best of author’s knowledge, this is the first attempt for filamentous sludge bulking prediction with multiple-step ahead prediction.

Different from traditional fault diagnosis and prognosis having decisions making exactly, uncertainty was involved to form the worst case for decision making. This approach is adopted by considering the uncertainty information from factors such as model parameters, collected data and the significant disturbances of a WWTP. Furthermore, ±15 mL/g was added to the limited filamentous bulking to make the fault diagnosis and prognosis more reliable to account for the uncertainty influence. Due to sufficient time requirement to deal with the filamentous bulking, not only one day ahead but also six days was presented. Although in the case study we limited the number of predicted horizon to six days, the methodology is clearly applicable when much more days ahead predictions are made. This will provide sufficient time for filamentous sludge bulking control.

However, since the recursive multi-step ahead prediction is performed, avoiding predicted error accumulation is infeasible. In fact, it only needs to improve the recursive way by combining the direct prediction methodology^27^. A direct prediction methodology aims to estimate a set of *H* prediction models, each returning a forecast for the *i*th values (*i *∈ {1, …, *H*}, where *H* represents the number of predicted step). The idea behind this strategy is to combine aspects from both, the direct and the recursive strategy. In other words, a different model is used at each step but the approximations from previous steps are introduced into the input set. We presented the methodology using an OD process as an example. The methodology can be extended to other activated sludge processes. The use of GPR model is able to be expanded to other models with uncertainty description. The performance of more powerful prediction algorithm requires further study.

In this study, we demonstrated the novel design of fault diagnosis and prognosis of filamentous sludge bulking through simulation studies. Our proposed model can track and predict the variations of SVI, but it cannot distinguish the bulking type (filamentous bulking or non-filamentous bulking). In order to distinguish the bulking type, integration of image analysis with model prediction will be performed further in future. While we used an advanced model giving realistic representations of real system, the proposed fault diagnosis and prognosis methodology requires further verification through its application into a real activated sludge process.

## Methods

### GPR model

GPR model is a simple and general class of models of functions. To be precise, a GPR is any distribution over functions such that any finite set of function values {*f*(*x*_1_), *f*(*x*_2_), …*f*(*x*_*n*_)} have a joint Gaussian distribution. GPR is usually formulated as follows: given a training set 
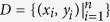
 of *n* pairs of inputs *x*_*i*_ and noisy outputs *y*_*i*_, compute the predictive distribution of *f* at a new testing input x*. We assume that the noise is additive, independent and Gaussian, such that the relationship between the (latent) function *f* (*x*_*i*_) and the observed noisy targets *y* are given by^28^


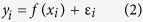



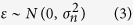



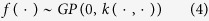


where 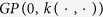 represents a Gaussian process with mean and covariance matrix equaling to 0 and *k*(·, ·), respectively. The noise *ε* follows the Gaussian distribution with mean 0 and covariance 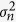.

To be simplicity, we define covariance matrix *K* = *k*_*ij*_. By inference, it is easy to obtain that the outputs follow multivariate joint Gaussian distribution:


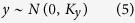


where 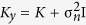, *K*_y_ is the covariance matrix with dimension being *n* × *n*, the corresponding (*i, j*)^*th*^ element is


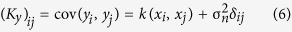


where *δ*_*ij*_ is the Kronecker function^29^. The major difference of *K* and *K*_*y*_ is that *K* is noise free but *K*_*y*_ is noise-depended. In summary, the parameters needed to be identified are formulated as 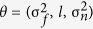, where *l* is the width of kernel *k*(*x*_*i*_, *x*_j_).

### OS ahead prediction with GPR

Due to 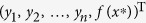 following Gaussian distribution also, the prediction at the location x^*^ can be obtained with the mean and variance:


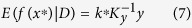



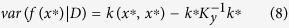


where 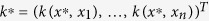
represents the correlated relationship between x^*^ and training set data *x*_*i*_. The corresponding likelihood function is shown as follows:


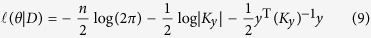


which can be optimized to derive *θ*. By performing OS prediction, the evolution of SVI can be monitored properly real time, even one sample ahead.

In the GPR model, the most commonly used covariance matrix is Squared-Exp (SE) shown as follows:


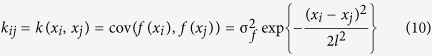


where 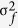 and *l* are hyper-parameters in need of identification, respectively. Different from the general Covariance function, we use a new proposed addictive Covariance function together with Squared-Exp (SE) as a base Covariance function shown as follows:


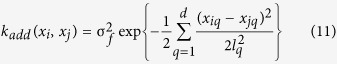


where 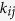 is the base kernel Squared-Exp (SE), *d* is the column number of training samples. This model, in fact, is a sum of functions of all possible combinations of input variables. This model can be specified by a weighted sum of all possible products of one-dimensional kernels. In our model, the only design choice necessary to specify an additive kernel is the selection of a one-dimensional base kernel for each input dimension. Parameters of the base kernels (such as length-scales (*l*_1_, *l*_1_, …, *l*_*d*_) can be learned as per usual by maximizing the marginal likelihood of the training data. It should be noted that commonly-used kernels such as the Squared-Exp (SE), Neural Network (NN) or Matérn kernels are local kernels^28^, depending only on the scaled Euclidean distance between two points. Therefore, models based on local kernels are particularly susceptible to the curse of dimensionality^15^, and are unable to extrapolate away from the training data. Methods based solely on local kernels sometimes require training examples at exponentially-many combinations of inputs. In contrast, additive kernels can allow extrapolation away from the training data, more details about kernel selection can be seen in Supplementary Information.

### MS ahead prediction with GPR

However, for most of cases, OS ahead prediction does not provide sufficient time for filamentous sludge bulking control. In order to predict time series values of many time steps into the future, MS techniques^30^,^31^ have been developed to achieve this goal. In this paper, we extend aforementioned GPR model for MS ahead prediction using a recursive strategy. In this strategy, a single model *f* is trained to perform one-step-ahead prediction firstly.


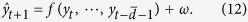


where 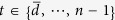. Sequentially, we use the value just forecasted as part of the input variables for estimating the next step with the same OS ahead model. This step is continued until the entire horizon, *H*, has been performed. Then the forecasts are given by:


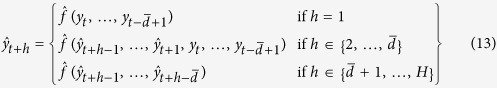


where *H* is the MS prediction horizon, 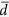 is the model input dimension, and 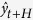 is an estimate of the output at time-step *t *+ *H*. Such the recursive strategy has been successfully used to forecast many real world time series by using different machine learning models^27^.

### Hybrid automata definition

Given the mixture of OS and MS prediction models in this paper, a tool to describe such hybrid behaviors is necessary. A hybrid automata provides an alternative to deal with this issue. The automaton is a formal model for a dynamic system with discrete and continuous components^32^. The semantics of a hybrid automaton is defined in terms of a labeled transition system between states, where a state consists of the current location of the automaton and the current valuation of the real variables. To formalize the semantics of the hybrid automaton, first we need to define the concept of a hybrid automaton’s state. Then, upon the constraint for each state, current valuation of the real variables can be obtained by a specified function or model. In fact, a hybrid automaton evolves depending on two kinds of transitions: continuous transitions, capturing the continuous evolution of states, and discrete transitions, capturing the changes of location^33^,^34^.

A hybrid automaton can be divided into autonomous and controlled types, which depend on whether their transitions are uncontrollable or controllable. By using the hybrid automata, the transition behaviors can be accounted for properly. More details for hybrid automata definition can be seen in the Supplementary Information. Also, an example to suggest how to construct a hybrid automaton is shown in the Supplementary Information.

### General methods for prediction model construction

In order to provide references for the proposed methodology of OS-GPR for fault diagnosis, Partial Least Squares (PLS) is used firstly. The purpose of PLS is to find a linear regression model by projecting the input variables *X* and the output variables *Y* to a new space. Because both *X* and *Y* data are projected to new spaces, the PLS family of methods are known as bilinear factor models. Secondly, a typical neural network Radial Basis Function (RBF)^35^,^36^ is considered as an alternative to approach nonlinear relationship between the input variables *X* and the output variables *Y*. The output of the network is a linear combination of radial basis functions of the inputs and neuron parameters. However, due to significant nonlinearity for modeling data, the predictions using shallow neural networks (the number of layers are less than three) deviate from the true values dramatically and failed to represent the data properly. Therefore, as a comparison, a deep neural network (DeepNN) is presented thirdly, which is five layers and trained by the deep learning algorithm^15^.

In order to compare with the MS-GPR model for fault prognosis, a ARMA model is presented. An ARMA model provide a parsimonious description of a (weakly) stationary stochastic process in terms of two polynomials, one for the auto-regression (AR) and the second for the moving average (MA). Given a time series data *X*_t_, the ARMA model is a tool for understanding and being able to predict future values in this series^37^.

## Additional Information

**How to cite this article**: Liu, Y. *et al.* Prediction of Filamentous Sludge Bulking using a State-based Gaussian Processes Regression Model. *Sci. Rep.* **6**, 31303; doi: 10.1038/srep31303 (2016).

## Supplementary Material

Supplementary Information

## Figures and Tables

**Table 1:** Selected variables for model inputs.

Model inputs for OS GPR		Model inputs for MS GPR	
Variables	Comments	Variables	Comments
DO	Dissolved Oxygen (mg/L)	SVI(t)	Current data
COD	Chemical Oxygen Demand (mg/L)	SVI(t-1)	Data for one-step delay
Qin	Flow Influent (m^3^/d)	SVI(t-2)	Data for two-steps delay
SRT	Sludge Retention Time (d)	SVI(t-3)	Data for three-steps delay
MLSS(Oxidation ditch)	Mixed Liquor Suspended Solids (mg/L)	SVI(t-4)	Data for four-steps delay
SV%(Oxidation ditch)	Settling volume	SVI(t-5)	Data for five-steps delay
SV%(recycle)	Settling volume	SVI(t-6)	Data for six-steps delay
Temperature	Temperaturevalue (^o^C)	SVI(t-7)	Data for seven-steps delay
