# Supplementary material for: Prediction of Filamentous Sludge Bulking using a State-based Gaussian Processes Regression Model
Source: Sci Rep. 2016 Aug 8;6:31303. doi: 10.1038/srep31303 (PMC4976347; doi:10.1038/srep31303)
Supplement: Supplementary Information [file srep31303-s1.docx]

**Prediction of Filamentous Sludge Bulking using a State-based Gaussian Processes Regression Model**

Yiqi Liu^1,2^, Jianhua Guo*^2^,Qilin Wang^2^, Daoping Huang^1^

^1^ School of Automation Science & Engineering, South China University of Technology, Wushang Road, Guangzhou 510640, China

^2^ Advanced Water Management Centre, The University of Queensland, St. Lucia, Brisbane QLD 4072, Australia.

**Corresponding author:** Jianhua Guo; Level 4 Gehrmann Building, Advanced Water Management Centre, The University of Queensland, St. Lucia, Brisbane, QLD 4072, Australia

P: +61 7 3346 3215; F: +61 7 3365 4726; E-mail: [j.guo@awmc.uq.edu.au](mailto:j.guo@awmc.uq.edu.au)

**Supplementary Material 1. Kernel selection**

A kernel (also called a covariance function) is a positive-definite function of two inputs , , and is defined as to represent the similarity between two objects. The most few basic kernels are shown in Fig.S1, suggesting that each covariance function is able to make a different set of assumptions about the function we wish to model. Even if the kind of structure is not expressed by any known kernel, kernels can be combined to create new ones with different properties (Fig.S1 Line 2 and Line 3).


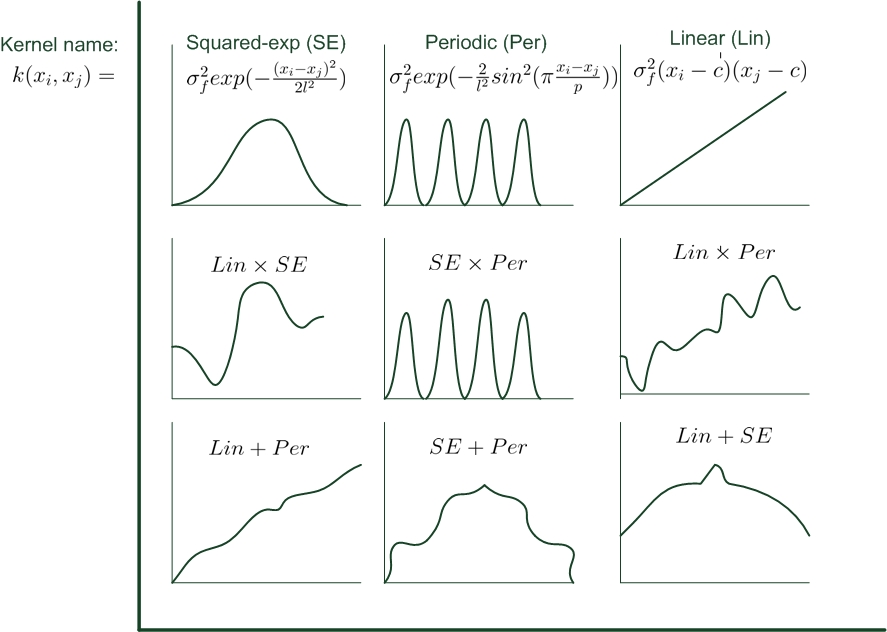


**Fig. S1.** Kernel functions for the GPR model

**Supplementary Material 2. HA definition**

An automaton is a formal model for a dynamic system with discrete and continuous components. A hybrid automaton is a tuple *H* = (*X, Q, Inv, Flow, E, Jump, Reset, Event, Init*) where:

- *X* is a finite set of *n* real-valued variables that model the continuous dynamics;
- *Q* is a finite set of control locations (mode);
- *Inv* is a mapping, which assigns an invariant condition to each location $q\in Q$. *Inv*(q) is a predicate over the variables in *X*. The control of a hybrid automaton remains at a location $q\in Q$, as long as *Inv*(*q*) holds;
- *Flow* is a mapping, which assigns a flow condition to each control location$q\in Q$. The flow condition Flow(*q*) is a predicate over *X* that defines how the variables in X evolve over the time *t* at location *q*;
- $E\subseteq Q\times Q$ is the discrete transition relation over the control locations;
- *Jump* is a mapping, which assigns a jump condition (guard) to each transition $e\in E$. The jump condition *jump*(e) is a predicate over *X* that must hold to fire *e*. Omitting a jump condition on a transition means that the jump condition is always true and it can be taken at any point of time. Conventionally, writing *Jump*(*e*)[*v*] means that the jump condition on a transition *e* holds, if the variations of variables on the transition *v*;
- *Reset*(*e*) is a predicate over *X* that defines how the variables are reset;
- *Event* is a finite set of events, and an edge labelling function *event*: $E\to\Sigma$ that assigns to each control switch an event;
- *Init* is the initial state of the automaton. It defines the initial location together with the initial values of the variables *X*.

**Supplementary Material 3. An example for hybrid automata model building**

To further illustrate hybrid automata, a pump station model based on autonomous hybrid automata is shown as follows. The hybrid automaton of Figure S2 models a pump station, which turns on and off according to the sensed water level. The variable *x* represents the water level. In control mode OFF, the pump station is off, and the water level rises according to the flow condition (*Flow*) , where *S* is pump station wet well area, *t* is the time, and *inflow* is incoming flow into pump station. In control mode ON, the pump station is on, and the water level falls or rises according to the Flow condition , where outflow is constant due to constant pump speed operation. In this example, initially, the pump station is off and the initialized water level is 0.5. According to the jump condition *x* >*UL* (water level upper limit)**,** the pump station may go ON as soon as the water level reaches *UL.*

Figure S2. Pump station autonomous hybrid automaton

According to the invariant condition (*Inv*) $x<UL$ in the OFF circle, the pump station will stay OFF when the water level is lower than *UL*. Similar behaviour will occur once water level is lower than *DL* (water level down limit) in the ON circle, if pump station is ON.
